# Control of Magnetism via *B*‑Site Order and Disorder in Y_2_NiTiO_6_ Perovskite

**DOI:** 10.1021/acs.inorgchem.5c03186

**Published:** 2025-10-06

**Authors:** Nataliya L. Gulay, Hai Lin, Anna Krowitz, Manel Sonni, Troy D. Manning, Luke M. Daniels, Matthew S. Dyer, John B. Claridge, Matthew J. Rosseinsky

**Affiliations:** † Department of Chemistry, Materials Innovation Factory, 4591 University of Liverpool, 51 Oxford Street, Liverpool L7 3NY, U.K.; ‡ Leverhulme Research Centre for Functional Materials Design, Materials Innovation Factory, 4591 University of Liverpool, 51 Oxford Street, Liverpool L7 3NY, U.K.

## Abstract

The first reported phase in the Y_2_O_3_–NiO–TiO_2_ chemical space, the Y_2_NiTiO_6_ perovskite undergoes a temperature-induced order–disorder transition. Above ∼1700 K, it adopts the structure of a disordered CaTiO_3_-type orthorhombic perovskite with *a* = 5.26939(2), *b* = 5.60367(2), and *c* = 7.58137(3) Å, with the *B* site uniformly occupied by 0.5Ni+0.5Ti. Below this temperature, Y_2_NiTiO_6_ adopts rock-salt ordering of the transition metals in a monoclinic unit cell (*a* = 5.26695(2), *b* = 5.60164(2), *c* = 7.57493(2) Å, β = 90.4940(2)°) with 0.9/0.1 ordering of the *B* site. Ordering of Ni and Ti changes the magnetic properties from spin-glass behavior in the orthorhombic phase to antiferromagnetic order (*T*_*N*_ = 17 K) for the monoclinic phase, while the optical properties of both phases remain unchanged across the transition.

## Graphical abstract

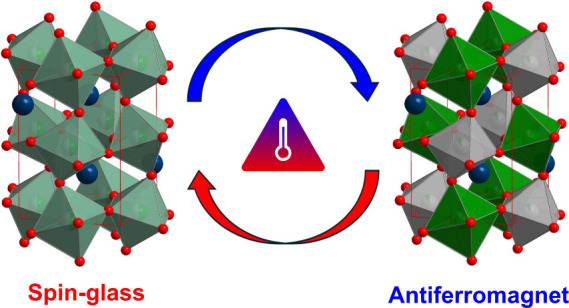

## 1 Introduction

Perovskites have been one of the most studied structural classes of materials with >37,000 entries in Pearson's Crystal Data database,^1^ which have been scrutinized during more than 185 years of research.^2^ In recent years, nickel- and titanium-containing perovskites attracted attention due to their potential as materials for hydrogen storage,^3^ solid oxide fuel cells,^4,5^ energy storage,^6^ and catalysis.^7,8^

Yttrium-based perovskites Y*B*O_3_ have also been considerably well studied. For example, YNiO_3_ attracted attention as it possesses interesting physical properties, namely, multiferroics with a metal-to-insulator transition at 580 K,^9–11^ related to a charge disproportionation of nickel resulting in two distinct *B*-site environments.^12^ YTiO_3_ is a Mott insulator with a ferromagnetic ground state.^13,14^ At the same time, the double perovskites Y_2_ *B′B″*O_6_ are much less studied (see Table SI1) than, for example, those reported for lanthanum-based ones.^15,16^

Among the perovskites listed in Table SI1, ordered double perovskites show a range of magnetic ordering, including paramagnetism to 5 K in high-pressure Y_2_CuTiO_6_,^17^ the antiferromagnets Y_2_NiRuO_6_ (T_N_ = 93 K)^18^ and Y_2_AlCrO_6_ (T_N_ = 81 K),^19^ Y_2_CoMnO_6_ that ferromagnetically orders below 76 K,^20^ and ferrimagnetic Y_2_MnCrO_6_ (T_C_ ∼ 75 K).^21^ However, some phases reveal the effects of the *B*-site disorder on the magnetic order. One example is the YNi*_*x*_* Mn_1–*x*_O_3_ system, where ordered *P*2_1_/*n* YNi_0.50_Mn_0.50_O_3_ is a ferromagnet, while disordered *Pbnm* YNi_0.25_Mn_0.75_O_3_ shows spin-glass behavior.^22,23^ Similarly, pressure-induced *B*-site disorder in Y_2_CoIrO_6_ and Y_2_CoRuO_6_ causes loss of long-range ferromagnetic ordering in these phases.^24^

Double perovskites with Ni and Ti have been observed so far only for La,^25^ Ho,^26^ and Sr^27^ on the *A*-site. At the same time, Ni and Ti ordering on the *B*-sites of double perovskite *A*_2_ *B′B″*O_6_ has been proven for Ho_2_NiTiO_6_ ^26^ and La_2_NiTiO_6_,^28^ and neutron diffraction studies were necessary to prove such cation ordering and subsequently monoclinic symmetry for the lanthanum compound.^28^ Variety of reports on structure and Ni/Ti ordering within La_2_NiTiO_6_ perovskite (Table SI2) highlights the necessity of accurate structure determination, as it underpins the understanding of the properties of the materials.

There have been no reports of the compounds forming in the Y_2_O_3_–NiO–TiO_2_ chemical space in the structural databases (Inorganic Crystal Structures Database^29^ and Pearson’s).^1^ There has been a study on Y^3+^-doped Ni–Ti–O ceramics, which, however, resulted in a mixture of binary and ternary phases.^30^ During the investigation of reactivity in the underexplored Y_2_O_3_–NiO–TiO_2_ system, we discovered a new Y_2_NiTiO_6_ perovskite that undergoes an order-disorder transition at ∼1700 K that is easily recognizable from powder X-ray diffraction (PXRD) data. Both phases were isolated by control of synthetic conditions, which allowed further examination and comparison of structures and properties at room temperature and below. Despite the similar color and optical properties, ordering of nickel causes a change in magnetic ordering at low temperatures. The synthesis, crystal structure, and magnetic properties of these perovskites are described herein.

## 2 Experimental Part

### 2.1 Synthesis

Y_2_NiTiO_6_ perovskites were formed by the solid-state reaction of oxide powders: yttrium­(III) oxide (Alfa Aesar, 99.999%), nickel­(II) oxide (Sigma-Aldrich, 99.99%), and rutile titanium­(IV) oxide (Sigma-Aldrich, 99.8%). The oxides were dried in a furnace at 493 K prior to weighing the stoichiometric quantities with respect to the Y_2_NiTiO_6_ final formula. The samples with a target weight of ∼0.5 g were ground in an agate mortar and pestle by hand, while the bigger batches of ∼2 g were prepared by ball-milling the starting reagents. A first round of reactions was run with the prereacted reagents pressed in pellets; however, further experiments with loose powders proved to provide the same yield, and the pressing step was omitted.

After the starting oxide mixtures were homogenized, they were placed in alumina crucibles and prereacted at 1273 K for 12 h to prevent potential NiO loss. The heating and cooling rates were kept at 5 K min^–1^ unless stated otherwise. The monoclinic Y_2_NiTiO_6_ forms if reacted at 1573 K and transforms into the orthorhombic phase above 1700 K. The best purity samples were obtained by placing the prereacted mixtures into zirconia crucibles and annealing at 1730 K for 24 h. The furnace was switched off after this treatment, which yielded a phase-pure polycrystalline sample of orthorhombic Y_2_NiTiO_6_. The prepared orthorhombic samples were used for further synthesis of the monoclinic phase by placing them into zirconia crucibles and annealing at 1573 K for 24 h for 4–6 times with intermediate regrinding and examination using PXRD analysis until no changes in diffraction patterns were spotted. The powders do not change their color upon the phase transition, and both monoclinic and orthorhombic Y_2_NiTiO_6_ are colored dark green.

### 2.2 Powder Diffraction

The in-house data for the routine PXRD analysis were collected on a PANalytical X’Pert diffractometer (monochromatic Co Kα1 radiation, λ = 1.78896 Å). The measurements of evenly spread polycrystalline samples on greased glass slides were performed in the reflection mode. For the phase analysis, the diffraction data were matched with patterns of known phases using X’Pert HighScore Plus software.

High-resolution synchrotron diffraction data were collected at Diamond Light Source, beamline I11, using a Mythen position-sensitive detector (PSD). For the measurement, the samples were loaded into borosilicate glass capillaries of 0.3 mm diameter. The measurements were recorded at room temperature (293 K). Structures of the Y_2_NiTiO_6_ perovskites were solved using TOPAS Academic V7.^31^ The bond valence sum (BVS) was determined using the GBondStr tool of the FullProf suite.^32^ Crystal structures were visualized using Diamond software (version 4.6.8).^33^

### 2.3 Energy Dispersive X-ray Spectroscopy

The composition of the samples was confirmed using energy dispersive X-ray spectroscopy (EDX) analysis integrated with a transmission electron microscope (TEM). The samples were dispersed on carbon-coated copper TEM grids and were inserted using a tomography holder and measured on a JEOL2100+ operating at 200 kV equipped with an SDD detector from Oxford Instruments (Model: X-Max 65T with a 65 mm^2^ surface area detection). Small, isolated particles were chosen for analysis to avoid collecting data from the agglomerates. Data acquisition and analysis were performed using Aztec software. Correction factors were determined by measuring corresponding standards for each chemical element.

### 2.4 Magnetic Properties Measurements

The Y_2_NiTiO_6_ samples were ground to fine powders; small quantities (∼10 mg) were weighed and placed in a polypropylene capsule that was secured within a plastic straw holder. This was attached to a sample holder rod and placed in a chamber of a commercial superconducting quantum interference device (SQUID) magnetometer from Quantum Design. For magnetic susceptibility, the data were recorded in a range of 2–300 K with an applied external magnetic field of 1 kOe in the field-cooled (FC) and zero-field-cooled (ZFC) modes. To capture the spin-glass behavior, a higher field of 10 kOe was applied to a sample prior to the temperature- and time-dependent measurements.

The data were fitted and plotted with Origin Pro 2024b software.

### 2.5 Diffuse Reflectance Measurements

Diffuse reflectance of the powders was measured using an Agilent Cary 5000 instrument between 200 and 2500 nm with a step size of 1 nm. Calibration to 100 and 0% reflectance was performed prior to measurement using a poly­(tetrafluoroethylene) (PTFE) standard and a light trap, respectively. The diffuse reflection (*R*) spectra were transformed using the Kubelka–Munk function, *F*(*R*) = (1 – *R*)^2^/2*R*, and fitted to a combination of 4 functions modeling intraband transitions (two Gaussian), indirect band gap (power law, *y* = *y*_0_ + *A* ^(*x*–*x*_*g*_)^0.5^^), and a disorder-induced tail (exponential, $y=y_{0}+Ae^{\left(-x/c\right)}$ ), where *x*_*g*_ is the position of the absorption edge; *c* is the decay constant for the Urbach tail, and *y*_0_ is a constant background; a variable *X*_c_ was used as a matching point between the Urbach tail and the indirect absorption edge.^34^ Fitting was performed using the nonlinear fitting tool of Origin Pro 2024b. Additionally, the band gap was determined from a Tauc plot using a method described by Makuła et al.^35^

### 2.6 Density Functional Theory Calculations

Periodic plane-wave-based density functional theory (DFT) calculations were performed on the fully ordered *P*2_1_/*n* structure of Y_2_NiTiO_6_ using the VASP package^36^ with the HSE06 hybrid density functional.^37^ A 600 eV plane-wave cutoff energy was used along with the projector augmented-wave method for the treatment of core electrons.^38,39^ Calculations were performed with both ferromagnetic and antiferromagnetic colinear magnetic ordering of the Ni ions. The unit cell and atomic positions were optimized until all forces fell below 0.01 eV Å^–1^. The band structure was calculated along a high-throughput path in reciprocal space as suggested by AFLOW.^40^

## 3 Results and Discussion

### 3.1 Structure of Y_2_NiTiO_6_

The structures of the orthorhombic and monoclinic Y_2_NiTiO_6_ were refined using TOPAS Academic V7.^31^ As starting models, the known structures of CaTiO_3_ (*Pbnm*)^41^ and YNiO_3_ (*P*2_1_/*n*)^12^ were exported from Pearson’s^1^ database and refined against high-resolution synchrotron diffraction data. For both higher and low temperature samples, a small broadening of the reflections was observed, which can be attributed to residual monoclinic and orthorhombic phases, respectively. Therefore, the best fit was obtained when including both perovskite phases in the refinement (see Figure 1a,b). In the case of the high-temperature orthorhombic sample, the occupancy of the mixed-occupied Ni/Ti site was freely refined to a 50:50 ratio within three times the standard deviation. For the monoclinic phase, the completely ordered model was tested, where Ni and Ti were assigned 2*d* and 2*c* atomic sites, respectively. However, this model yielded a large discrepancy in thermal parameters for these positions. Therefore, each site was assigned to mixed Ni/Ti occupancy, and the occupancies were constrained to yield a 1:1 ratio of Ni to Ti in the phase overall, as confirmed by EDX (see Figure SI2). The mixed sites *M*1 and *M*2 were refined to contain 0.91(1)Ti + 0.09(1)Ni and 0.90(2)Ni + 0.10(2)­Ti, respectively, and resulted in uniform positive thermal parameters. The occupancy of the Y site was also freely refined to account for possible deficiency, but it resulted in a value of 0.996(4) of yttrium and therefore was set to unity in the final refinement. The refinement details are given in Table 1, while the refined atomic coordinates and the interatomic distances are listed in Tables SI3 and SI4, respectively. The refined compositions of Y_2_Ni_0.99(2)_Ti_1.01(2)_O_6_ for monoclinic and Y_2_Ni_1.04(2)_Ti_0.96(2)_O_6_ for orthorhombic phases are charge-balanced, correlate well with each other, and are in a good agreement with experimental EDX data within three times standard deviation (average of Y 1.70(8): Ni 1.05(5): Ti 1.00(4) and Y 1.9(1): Ni 1.02(8): Ti 1.00(6) for the orthorhombic and monoclinic phases, respectively, Figure SI2).

**Figure 1:**
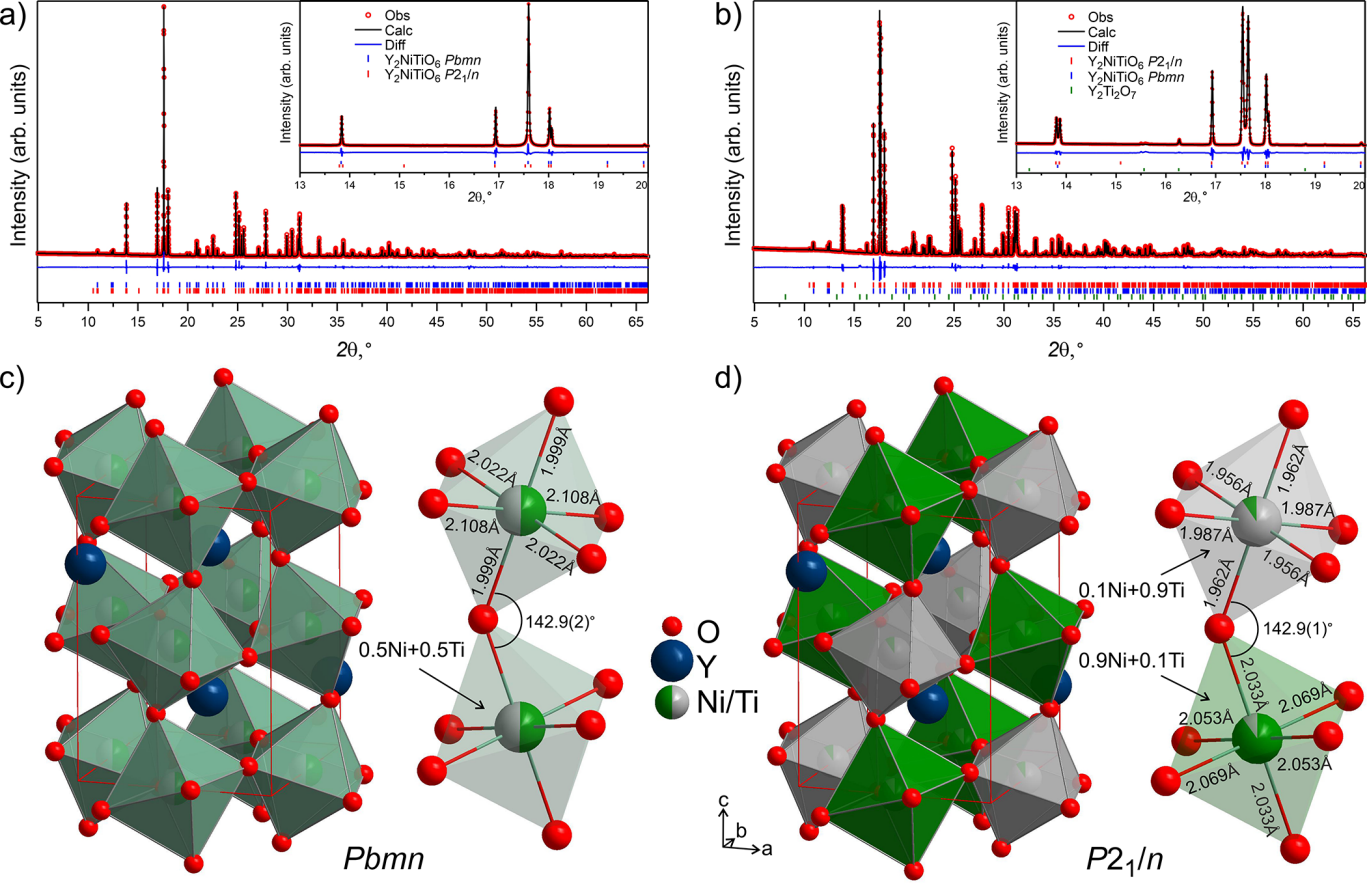
Top: Rietveld refinement of synchrotron PXRD (I11, Diamond Light Source, PSD detector, λ = 0.824556 Å) collected at room temperature for Y_2_NiTiO_6_ perovskites. Insets showed an enlarged region from 13 to 20 2θ that shows the presence or absence of peak splitting. Observed, calculated, and the difference intensity are drawn in red circles, black line, and blue line, respectively. (a) Sample with Y_2_NiTiO_6_-orthorhombic perovskite (*Pbmn*) as a main phase (92.6(2) wt%) and 7.4(2) wt% of Y_2_NiTiO_6_-monoclinic (*P2*_*1*_ */n*). (b) Sample with Y_2_NiTiO_6_-monoclinic perovskite (*P2*_*1*_ */n*) as a main phase (91.9(1) wt%), 6.8(1) wt% of Y_2_NiTiO_6_-orthorhombic (*Pbmn*) and 1.21(2) wt% of Y_2_Ti_2_O_7_. Bragg reflections for Y_2_NiTiO_6_-orthorhombic, Y_2_NiTiO_6_-monoclinic, and Y_2_Ti_2_O_7_ are shown as blue, red, and green ticks, respectively. Bottom: Structures of the Y_2_NiTiO_6_ perovskites were projected along the *c*-axis. (c) Disordered Y_2_NiTiO_6_-orthorhombic perovskite (*Pbmn*). (d) Mostly ordered Y_2_NiTiO_6_-monoclinic perovskite (*P*2_1_/*n*). For the Ni/Ti@O_6_ octahedra, relevant interatomic distances and angles are highlighted to showcase the change of the coordination environments with ordering. Yttrium, nickel, titanium, and oxygen atoms are drawn as dark-blue, green, gray, and red spheres, respectively.

**Table 1:** Crystallographic Data and Refinement Details for Y_2_NiTiO_6_ Perovskites from Rietveld Refinements of the Synchrotron PXRD Data (λ = 0.824556 Å) Collected at Room Temperature

phase	Y_2_NiTiO_6_-orthorhombic	Y_2_NiTiO_6_-monoclinic
refined composition	Y_2_Ni_1.04(2)_Ti_0.96(2)_O_6_	Y_2_Ni_0.99(2)_Ti_1.01(2)_O_6_
formula weight, g mol^–1^	380.75(18)	379.5(7)
space group	*Pbnm*	*P*2_1_ */n*
*Z*	2	2
lattice parameters, Å	*a* = 5.26939(2)	*a* = 5.26695(2)
	*b* = 5.60367(2)	*b* = 5.60164(2)
	*c* = 7.58137(3)	*c* = 7.57493(2)
		β = 90.4940(2)°
cell volume, Å^3^	223.836(2)	223.4788(11)
density, g cm^–3^	5.649(3)	5.640(10)
2θ, ° range	4–66.446	4–66.446
2θ, ° step	0.004	0.004
no. of refined parameters	47	71
*R*_*p*_ *,* %	5.05	3.19
*R*_*wp*_ *,* %	7.83	4.79
*R*_*exp*_ *,* %	0.93	0.62
*R*_*Bragg*_ *, %*	2.86	1.53
GOF (*R*_*wp*_/*R*_*exp*_)	8.46	7.73

Y_2_NiTiO_6_ perovskite is the first quaternary compound in the Y_2_O_3_−NiO−TiO_2_ phase field. Above 1700 K, Y_2_NiTiO_6_ crystallizes as a disordered orthorhombic perovskite, space group *Pbnm*, with a pronounced octahedral tilting (*a* ^+^ *b* ^–^ *b* ^–^ according to Glazer notation).^42,43^ Its structure is drawn in Figure 1c. After lowering the temperature below 1670 K, the structure undergoes a common rock-salt ordering in space group *P*2_1_/*n* (*a* ^+^ *b* ^–^ *c* ^–^ according to Glazer notation).^42,43^ The structure of monoclinic Y_2_NiTiO_6_ is shown in Figure 1d. Curiously, such ordering transition is not exclusive for double perovskites; for example, it has been observed for YNiO_3_, where it is driven by a charge disproportionation.^12^

The unit cell of orthorhombic Y_2_NiTiO_6_ is slightly distorted compared to the idealized cubic cell (*a*:*b*:c/ $\sqrt{2}$ = 1:1.063:1.017). Surprisingly, the unit cell volume of Y_2_NiTiO_6_ remains mostly unchanged after the ordering (see Table 1), and only the β angle increases up to 90.4940(2)°, which is much higher than that for monoclinic YNiO_3_ (β = 90.0806(2)°).^11^ At the same time, the *B*-site ordering is evident from two distinct types of octahedra in the structure of monoclinic Y_2_NiTiO_6_: smaller *M*1@O_6_ occupied mainly by titanium (0.91(1)Ti + 0.09(1)­Ni) with *M*1–O distances ranging from 1.956 to 1.987 Å and larger *M*2@O_6_ dominated by nickel (0.90(2)Ni + 0.10(2)­Ti) with *M*2–O distances ranging from 2.033 to 2.070 Å. These interatomic distances correlate well with those observed for pure titanium- and Ni-centered octahedra (e.g., 1.946 Å in CaTiO_3_ ^41^ and 1.981–2.026 Å for a larger Ni@O_6_ in YNiO_3_)^12^ as well as for the ordered La_2_NiTiO_6_ (1.933–1.990 Å for Ti–O and 2.032–2.071 Å for Ni–O).^28^ Along the *c* axis, the tilt angle between the octahedra remains mostly unchanged upon the transition; however, the ordering affects the corresponding angles in the *ab* plane (144.4(1)° for orthorhombic; 144.2(1)° and 145.5(1)° for monoclinic phase, see Figure SI1). For both variants of Y_2_NiTiO_6_, the *B*–O–*B* angles are much smaller than those observed for La_2_NiTiO_6_ (155.3–161.4°),^28^ which indicates a higher degree of octahedral tilting in the structure of Y_2_NiTiO_6_ to accommodate the smaller yttrium cations.

The *A*-site coordination in the structure of Y_2_NiTiO_6_ remains mostly unchanged upon ordering (Table SI5). Yttrium is coordinated by 8 oxygen atoms, and the distances to the 6 closest of them change by no more than 0.013 Å after the phase transition. The pair of longest Y–O distances in the structure of the orthorhombic phase (2.677 Å) changes more significantly (2.647 and 2.702 Å), corresponding to smaller and larger octahedra in the monoclinic perovskite. However, even the longest distances remain within the range observed for other yttrium oxide compounds, e.g., 2.222–2.684 Å for YTiO_3_.^44^ In the structure of La_2_NiTiO_6_, the corresponding La–O distances range from 2.420 to 2.758 Å.^28^

### 3.2 Density Functional Theory

The DFT optimized structure has lattice parameters close to the experimentally refined structure (*a* = 5.26, *b* = 5.60, *c* = 7.56 Å, β = 90.8°). Monoclinic double perovskites can show 3 types of antiferromagnetic orders: Type I and Type II, which are dominated by near-neighbor (NN) and next-near-neighbor (NNN) interaction of magnetic ions, respectively, and Type III with prevailing NN coupling in the presence of significant NNN interactions.^28^ Direct determination of the antiferromagnetic ordering would be possible from neutron diffraction data. We compared the DFT Type I and Type III magnetic orderings of Y_2_NiTiO_6_ and found that Type III is only 0.7 meV/Ni more stable than Type I, indicating that the two magnetic orderings are energetically competitive. Partial density of states plots show no significant differences for the two computed types (Figure SI3). In turn, Type I antiferromagnetic ordering of Ni is found to be 1.5 meV/Ni more stable than ferromagnetic ordering, suggesting a small exchange interaction and that any magnetic ordering will occur only at low temperatures. The small energy differences show that more work is needed to identify the true AFM ground state. DFT calculation of Type II ordering requires an even larger supercell than Type III and would be prohibitively computationally expensive.

An indirect charge transfer gap of 4.27 eV is calculated from the valence band maximum at the C point in reciprocal space to the conduction band maximum at the Γ point for Type I ordered Y_2_NiTiO_6_ (Figure 2).

**Figure 2:**
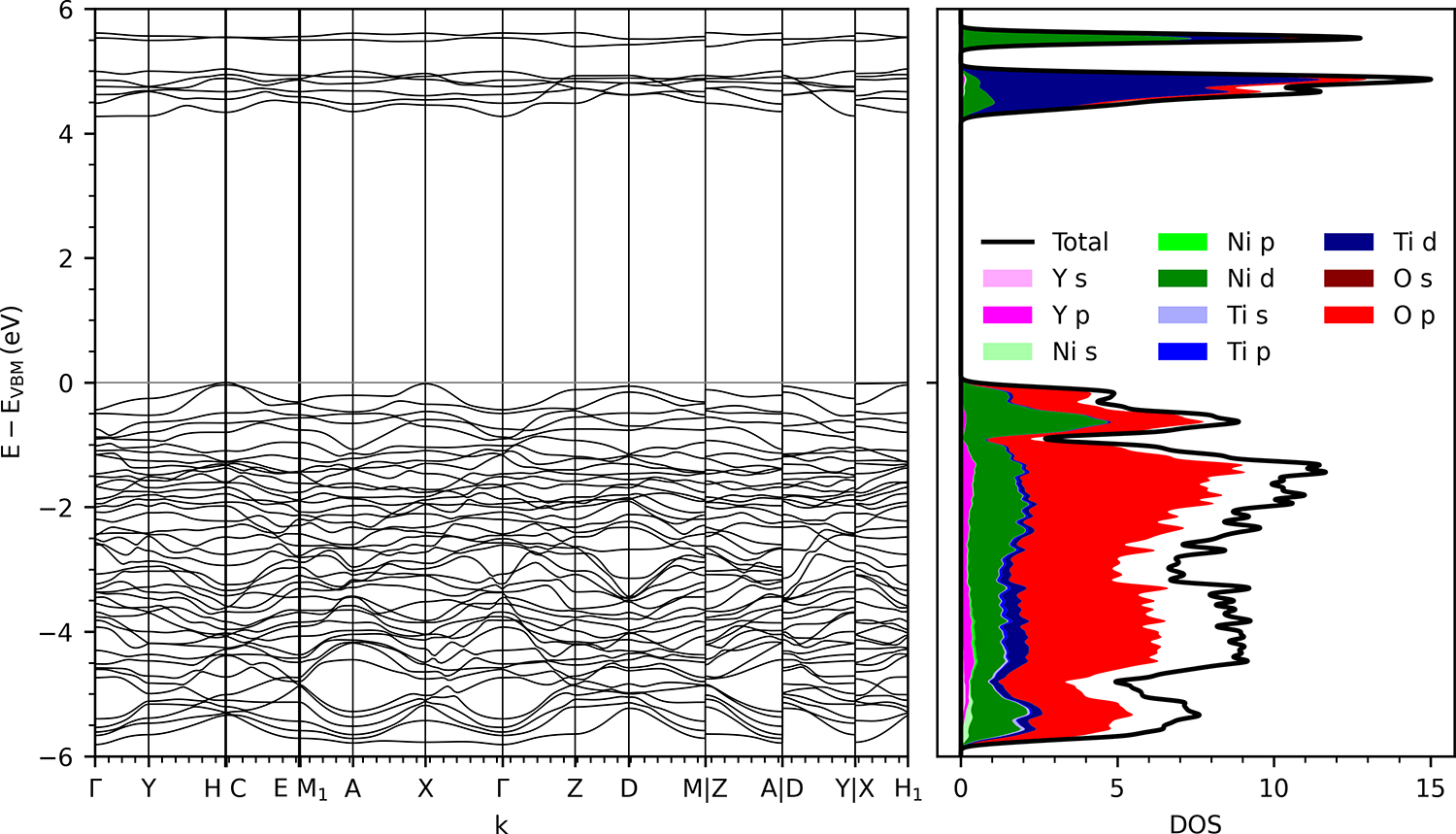
DFT band structure (left) and partial density of states (right) of Type I antiferromagnetically ordered Y_2_NiTiO_6_ in the fully site-ordered *P*2_1_/*n* structure.

### 3.3 Optical Properties

In contrast to the black color of La_2_NiTiO_6_,^45^ both orthorhombic and monoclinic Y_2_NiTiO_6_ have a dark-green color, which is consistent with the color associated with Ni^2+^ ions in aqueous solutions.^46^ The absorbance spectra (Figure 3a) for these materials feature three prominent absorption bands which correspond to those characteristic for the Ni^2+^ compounds at 400, 800, and 1400 nm, corresponding to ^3^A_2g_ → ^3^T_1g_(F), ^3^A_2g_ → ^3^T_1g_(G), and ^3^A_2g_ → ^3^T_2g_ transitions, respectively.^47^

**Figure 3:**
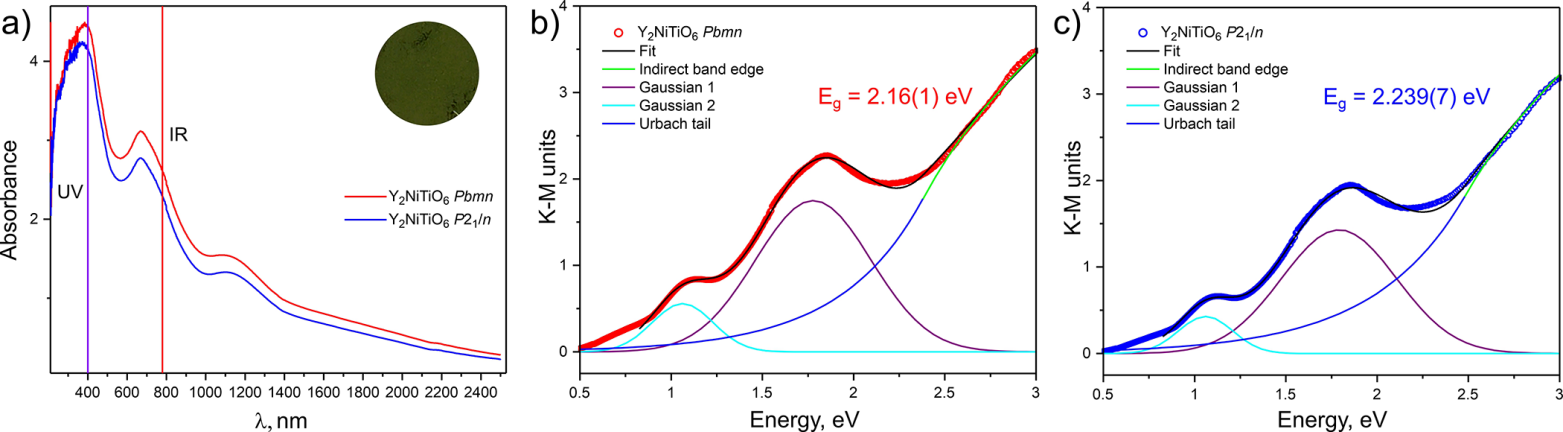
(a) Absorbance spectra of orthorhombic (red) and monoclinic (blue) Y_2_NiTiO_6_ perovskites. Inset: color of the powders (photo). Kubelka-Munk transformed diffuse reflection spectra of orthorhombic (b) and monoclinic (c) Y_2_NiTiO_6_ perovskites were fitted to a combination of 4 functions (see Table SI6 for fitting parameters) to model two most prominent visible light transitions, disorder-induced Urbach tail, and an indirect band edge. The values of the band edge correlate well with those obtained from the Tauc plot (Figure SI3).

To extract the values of band gap, the Kubelka–Munk transformed diffuse reflection spectra for both phases were fitted with two Gaussian functions corresponding to the most prominent peaks, an exponential function for the Urbach tail, and a power-law-dependent band edge (fitting details are listed in Table SI6).^48^ The orthorhombic and monoclinic Y_2_NiTiO_6_ are characterized with a band gap of 2.16(1) and 2.239(7) eV, respectively, and these values correlate well with those estimated from the Tauc plot (Figure SI4). The lowest energy observed optical transitions (Figure 4) are substantially lower in energy than the charge transfer gap calculated by DFT. It is likely that they arise from Frenkel exciton *d*-*d* transitions similar to those observed in NiO^49,50^ and only accessible computationally through a more complete consideration of electronic excited states.^51^ Similar values of band gap were observed for other Ni^2+^ compounds, e.g., NiTiO_3_ (2.2–2.5 eV).^52^

**Figure 4:**
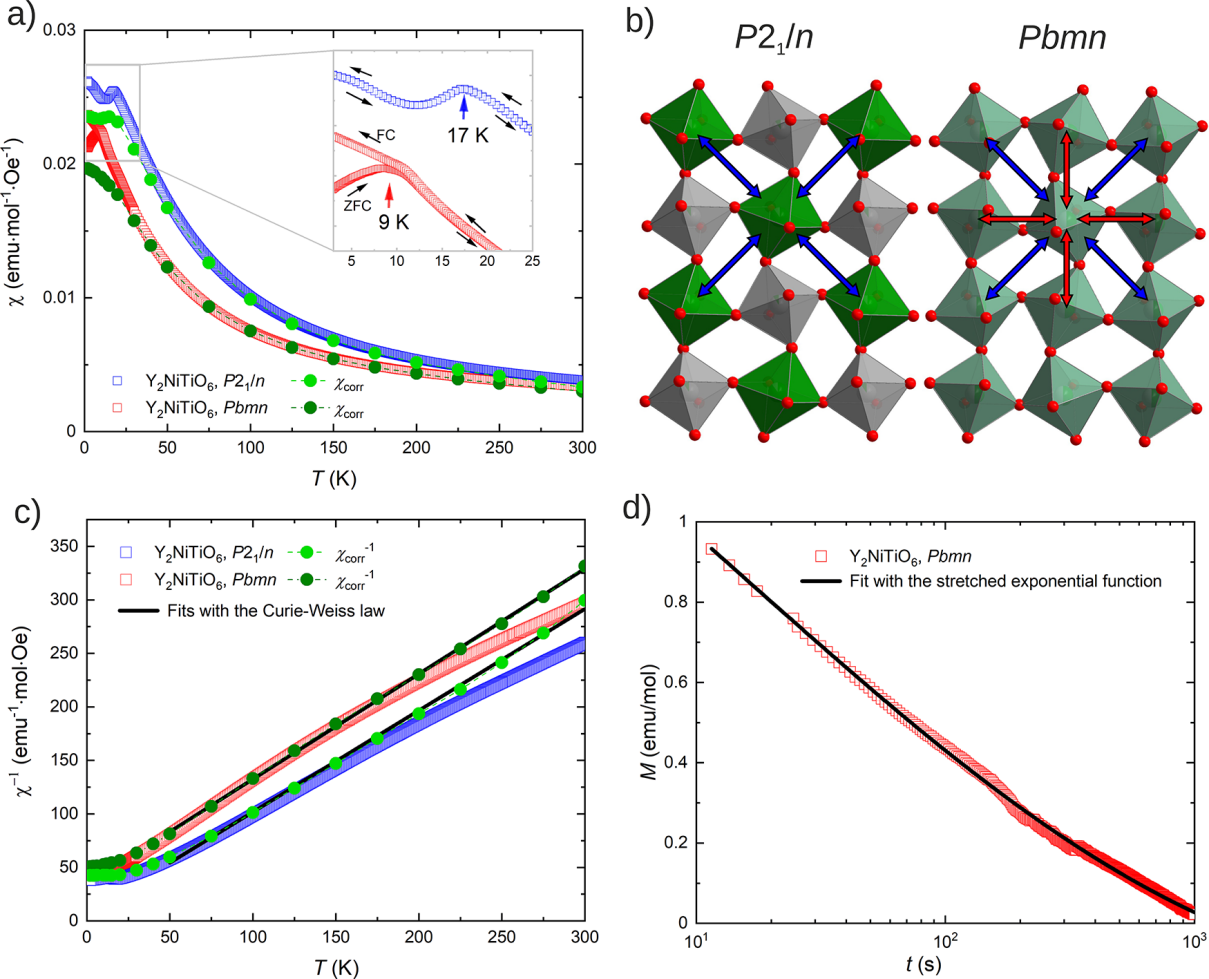
Magnetic properties of orthorhombic (red) and monoclinic (blue) Y_2_NiTiO_6_ perovskites. (a) Temperature dependence of magnetic susceptibility χ in the external magnetic field of 1000 Oe recorded upon heating after zero-field-cooling and field-cooling, respectively. The inset shows an enlarged view of the antiferromagnetic transition for monoclinic Y_2_NiTiO_6_ and the spin-glass behavior for orthorhombic Y_2_NiTiO_6_. The dark and light green points represent the corrected paramagnetic susceptibility χ_corr_ for orthorhombic and monoclinic Y_2_NiTiO_6_, respectively, obtained by the Honda-Owen method from the linear parts of the χ­(1/*H*) plots under high magnetic fields (4 – 7 T). (b) *B*-site cation distribution showing the Ni–Ni interactions in two phases. The blue arrows mark the diagonal nearest-neighbor and the next-nearest-neighbor Ni–Ni interactions in monoclinic and orthorhombic Y_2_NiTiO_6_, respectively. The red arrows mark the nearest-neighbor Ni–Ni interaction in the Ni-clustering zone of orthorhombic Y_2_NiTiO_6_, due to the Ni/Ti disorder. The competition between two different types of interactions can cause the spin-glass behavior. (c) Inverse magnetic susceptibility and fits with the Curie-Weiss law (black lines). Due to the slight curvature, its fit is performed on the corrected paramagnetic susceptibility χ_corr_^–1^ (dark and light green points). (d) Time decay of the magnetization for the orthorhombic Y_2_NiTiO_6_ in a logarithmic plot. The black line shows the fit with the stretched exponential function.

### 3.4 Magnetic Properties

The temperature dependences of magnetic susceptibility for both orthorhombic and monoclinic Y_2_NiTiO_6_ measured upon heating after ZFC and FC, respectively, are shown in Figure 4a. Both materials behave as Curie-Weiss paramagnets in a broad range of 50–300 K with room temperature magnetic susceptibilities of 3.36 × 10^–3^ and 3.84 × 10^–3^ emu/mol for orthorhombic and monoclinic Y_2_NiTiO_6_, respectively. At low temperature, monoclinic Y_2_NiTiO_6_ shows an antiferromagnetic ordering with *T*_*N*_ of 17 K. This correlates well with the ordering predicted with DFT. In contrast, orthorhombic Y_2_NiTiO_6_ behaves as a spin-glass with the freezing temperature *T*_*f*_ of 9 K, revealed by the irreversible divergence between the ZFC and FC branches (Figure 4a, inset). This correlates with a higher degree of Ni–Ti disorder in the structure. In the rock-salt-ordered monoclinic phase, the diagonal nearest-neighbor Ni–Ni exchange interaction (marked with blue arrows in Figure 4b) is dominant and antiferromagnetic.^53^ In contrast, the Ni/Ti disorder in the orthorhombic phase can create zones with clustering Ni^2+^ ions that introduce additional possibilities for Ni–Ni interactions (red arrows in Figure 4b).^53^ The introduction of such interactions leads to competing magnetic orders and thus a spin-glass behavior. The spin-glass behavior arising from competing interactions due to site disorder has been demonstrated in other double perovskites such as YNi*_*x*_* Mn_1–*x*_O_3,_ ^22,23^ Y_2_CoIrO_6_,^24^ and Sr_2_FeTeO_6_,^54^ albeit the nature of these interactions is different from those in Y_2_NiTiO_6_.

The χ^–1^ plots (Figure 4c) for both orthorhombic (red points) and monoclinic (blue points) phases show slight curvature at high temperatures. Similar positive deviation from the Curie-Weiss law has been observed for the double perovskite Ba*_*x*_* Sr_2–*x*_TiCoO_6_ with the disordered *B*-site, and also the spin-glass behavior.^55^ This can be caused by a small quantity (below the detection limit of PXRD) of ferromagnetic impurities,^56^ such as residual Ni or NiO.^57^ Hence, instead of directly using the Curie-Weiss law, the Honda-Owen method^58–60^ was applied to extract the intrinsic paramagnetism and the impurity content from the ferromagnetic background. This method extrapolates the measured susceptibility

$$\chi =\chi_{\mathrm{corr}}+C_{\mathrm{sat}}M_{\mathrm{sat}}/H\;\mathrm{for}\;1/H\to 0$$

where χ_corr_ is the corrected susceptibility, *C*_sat_ is the presumed ferromagnetic impurity content, and *M*_sat_ is its saturation magnetization. Here, the *C*_sat_ *M*_sat_ values are 1.7(3) and 1.4(4) emu/mol at room temperature for the two phases, respectively. This *C*_sat_ would be expected to be a minute impurity content, of the order of 80 μg/g for Ni^61^ (*M*_sat_ = 55 emu/g), or 40 μg/g for NiO^57^ (*M*_sat_ = 105 emu/g). χ_corr_ can be obtained from the *y*-intercept of linear extrapolation of the χ­(1/*H*) plot between 4 and 7 T (see Figure SI5 for details). The resulting corrected susceptibilities χ_corr_^–1^ (Figure 4c) presented by dark and light green points for orthorhombic and monoclinic Y_2_NiTiO_6_, respectively, are both linear at high temperatures. The Curie-Weiss fits^56^ between 50 and 300 K are consequently performed on χ_corr_:

$${\chi_{\mathrm{corr}}}^{-1}=(T-T_{\vartheta })/C$$

where *C* is the Curie constant and *T*_ϑ_ the Weiss temperature. The effective magnetic moments $\mu_{\mathrm{eff}}=\sqrt{8C}$ are 2.85(1) μ_B_ for the orthorhombic phase and 2.95(2) μ_B_ for the monoclinic phase, which are close to the expected magnetic moment of 2.83 μ_B_ for Ni^2+^ observed in other octahedral Ni^2+^ oxides.^56^ For the monoclinic phase, the yielded *T*_ϑ_ is −13(2) K, of which the absolute value is close to its antiferromagnetic transition temperature *T*_*N*_ of 17 K. For the orthorhombic phase showing the spin-glass behavior, the yielded *T*_ϑ_ of – 34(2) K is also negative, signaling the dominant antiferromagnetic correlations within the magnetic frustration.^62^ Its frustration index f = *T*_ϑ_/*T*_*f*_ = 3.7 is smaller than that of a typical frustrated magnetic system (*f* > 5), indicating a relatively weak magnetic frustration in the orthorhombic phase.^56^

To characterize the glassy spin dynamics in orthorhombic Y_2_NiTiO_6_, the time decay of the magnetization is measured at 2 K, immediately after quickly turning off the external magnetic field of 10 kOe. As shown in Figure 4d, the magnetization decays slowly with time below the freezing temperature, signaling a relaxation process for typical spin glasses. The time decay can be fitted well by a stretched exponential function^63,64^

$$M(t)=M_{0}+M_{g}\exp\left[-{\left(\frac{t}{\tau_{r}}\right)}^{\beta }\right]$$

where *M*_0_ is the intrinsic magnetization, *M*_*g*_ the glassy component, τ_*r*_ the characteristic relaxation time constant, and β the stretching exponent, which is temperature dependent only between 0 and 1. The yielded τ_*r*_ is only 14(3) s, much smaller than those in the *R*NiO_2_ (*R* = La, Pr, Nd) nickelates with canonical spin-glass ground states (>1000 s).^65^ This indicates relatively hard spin dynamics in the disordered orthorhombic Y_2_NiTiO_6_.

The possibility of isolating two perovskite phases and studying their properties allows us to draw a connection between structural ordering and physical behavior. In the case of Y_2_NiTiO_6_, the structural ordering of the *B*-sites directly translates into the antiferromagnetic ordering of the monoclinic phase. Among double perovskites, such trends have been observed for PbIn_0.5_Nb_0.5_O_3_ ^66^ and PbSc_0.5_Nb_0.5_O_3_ ^67^ where the ordering degree played a significant role in the relaxor behavior of these phases, and for Sr_2_FeMoO_6_, with different magnetoresistance responses for ordered and disordered phases.^68^

## 4 Conclusions

In this work, we describe the synthesis and characterization of the first compound in the Y_2_O_3_−NiO−TiO_2_chemical space, the perovskite Y_2_NiTiO_6_ that undergoes an order–disorder transition at ∼1700 K. By controlling synthetic conditions, we were able to isolate ordered and disordered phases that allow their structural and magnetic characterization. The Ni/Ti rock-salt ordering on the *B*-site results in two distinct octahedral environments for these atoms, while the coordination of yttrium at the *A*-site remains unchanged. Analysis of the ordered monoclinic and disordered orthorhombic phases provides insight into the magnetic properties of these phases. The disordered Y_2_NiTiO_6_ shows spin-glass behavior at low temperatures; however, upon ordering, it orders antiferromagnetically at 17 K, which aligns with the DFT calculations.

## Supplementary Material



## Notes

Additional information on double perovskites with Y, Ni, and Ti; additional crystallographic information for orthorhombic and monoclinic Y_2_NiTiO_6_; and additional physical properties data (PDF)
