# Supplementary material for: Control of Magnetism via B‑Site Order and Disorder in Y2NiTiO6 Perovskite
Source: Inorg Chem. 2025 Oct 6;64(41):20705–13. doi: 10.1021/acs.inorgchem.5c03186 (PMC12541694; doi:10.1021/acs.inorgchem.5c03186)
Supplement: Supplementary file 1 [file ic5c03186_si_001.pdf]

Control of magnetism via *B*-site order and disorder in Y_2NiTiO_6 perovskite

Authors:

Nataliya L. Gulay[1], Hai Lin[1], Anna Krowitz[1], Manel Sonni[1], Troy D. Manning,[1] Luke M. Daniels[1], Matthew S. Dyer[1], John B. Claridge[1], Matthew J. Rosseinsky[1,2]

1. Department of Chemistry, Materials Innovation Factory, University of Liverpool, 51 Oxford Street, Liverpool, L7 3NY, UK

2. Leverhulme Research Centre for Functional Materials Design, Materials Innovation Factory, University of Liverpool, 51 Oxford Street, Liverpool L7 3NY, UK.

Supplementary information

Table SI1. List of reported $Y_2B'B''O_6$ phases that crystallize as ordered perovskite with $P2_1/n$ space group.

$Y_2B'B''O_6$	*B'*:*B*" ordering	Ref.
Y_2Mg**Ti**O_6	1:1	1
Y_2Zn**Ti**O_6	1:1	2
Y_2Cu**Ti**O_6	0.70/0.30	3
Y_2**Ni**RuO_6	0.600/0.400	4
Y_2**Ni**MnO_6	1:1	5
Y_2CoIrO_6	0.927/0.073	6
Y_2CoRuO_6	0.92/0.08	6
Y_2CoMnO_6	0.914/0.082	7
Y_2AlCrO_6	1:1	8
Y_2CrMnO_6	1:1	9

Table SI2. List of reported $A_2Ni_xTi_{1-x}O_6$ perovskites (searched though Pearsons' Crystal Data[10], selecting "perovskite" as structure class, Ni, Ti, and O in the composition, 4 elements in total).

$A_2Ni_xTi_{1-x}O_6$	Space group	Ni:Ti ordering	Ref.
$La_2Ni_{1-x}Ti_xO_6$ (x = 0 – 0.8)	$R3m$	disordered	11
$La_2Ni_{1-x}Ti_xO_6$ (x = 0.15 – 0.5)	$Pbnm$	disordered	12
$LaNi_{0.5}Ti_{0.5}O_3$	$P2_1/n$	ordered	13
$La_{2-x}NiTiO_{6-\delta}$ (x = 0 – 0.2)	$P2_1$	ordered	14
$La_2Ni_{1-x}Ti_xO_6$ (x = 0 – 0.15)	$R\bar{3}c$	disordered	15
$SrTi_{1-x}Ni_xO_3$ (x = 0 – 0.15)	$Pm\bar{3}m$	disordered	16

Table SI3. Site coordinates and isotropic thermal parameters for Y_2NiTiO_6 perovskites from Rietveld refinements of the synchrotron PXRD data (λ = 0.824556 Å) collected at room temperature.

Y_2NiTiO_6-orthorhombic, *Pbnm*						Y_2NiTiO_6-monoclinic, *P*2₁/*n*					
Site	Wyck.	*x/a*	*y/b*	*z/c*	B_{iso}, Å^2	Site	Wyck.	*x/a*	*y/b*	*z/c*	B_{iso}, Å^2
O1	4*c*	0.1119(5)	0.5430(5)	1/4	0.11(6)	O1	4*e*	0.1123(4)	0.5410(3)	0.2457(3)	0.21(5)
O2	8*d*	0.8103(4)	0.1975(4)	0.0582(3))	0.62(5)	O2	4*e*	0.8136(4)	0.2030(4)	0.0602(3)	0.14(5)
						O3	4*e*	0.6990(4)	0.6898(4)	0.0548(3)	0.09(5)
Y	4*c*	0.5184(1)	0.42925(7)	1/4	0.77(1)	0.996(4)Y	4*e*	0.51875(8)	0.42953(5)	0.24884(7)	0.302(8)
M: 0.482(8)Ti + 0.518(8)Ni	4*b*	1/2	0	0	0.66(2)	*M*1: 0.91(1)Ti +0.09(1)Ni	2*c*	0	1/2	0	0.16(3)
						*M*2: 0.90(2)Ni +0.10(2)Ti	2*d*	1/2	0	0	0.15(2)

Table SI4. Complete list of interatomic distances (Å) for the atoms in the structure of Y_2NiTiO_6 -orthorhombic perovskite (*Pbmn*) and Y_2NiTiO_6-monoclinic perovskite (*P2₁/n*) from Rietveld refinements of the synchrotron PXRD data (λ = 0.824556 Å) collected at room temperature. The occupancies for the mixed sites are: *M*: 0.482(8)Ti + 0.518(8)Ni for Y_2NiTiO_6-orthorhombic perovskite (*Pbmn*); *M*1: 0.91(1)Ti +0.09(1)Ni and *M*2: 0.90(2)Ni +0.10(2)Ti for Y_2NiTiO_6-monoclinic perovskite (*P2₁/n*).

Y_2NiTiO_6-orthorhombic, Pbnm				*Y_2NiTiO_6-monoclinic, P2₁/n*			
Atoms			*d*, Å	Atoms			*d*, Å
O1	2x	*M*	1.999(1)	O1	1x	*M*1	1.961(2)
	1x	Y	2.235(3)		1x	*M*2	2.033(2)
	1x	Y	2.271(3)		1x	Y	2.230(2)
					1x	Y	2.284(2)
O2	1x	*M*	2.017(2)	O2	1x	*M*1	1.987(2)
	1x	*M*	2.023(2)		1x	*M*2	2.053(2)
	1x	Y	2.278(2)		1x	Y	2.279(2)
	1x	Y	2.483(2)		1x	Y	2.470(2)
	1x	Y	2.677(2)		1x	Y	2.706(2)
				O3	1x	*M*1	1.956(2)
					1x	*M*2	2.070(2)
					1x	Y	2.283(2)
					1x	Y	2.487(2)
					1x	Y	2.647(2)
Y	1x	O1	2.235(3)	Y	1x	O1	2.230(2)
	1x	O1	2.271(3)		1x	O2	2.279(2)
	2x	O2	2.278(2)		1x	O3	2.283(2)
	2x	O2	2.483(2)		1x	O1	2.284(2)
	2x	O2	2.677(2)		1x	O2	2.470(2)
					1x	O3	2.487(2)
					1x	O3	2.647(2)
					1x	O2	2.706(2)
M	2x	O1	1.999(1)	*M*1	2x	O3	1.956(2)
	2x	O2	2.017(2)		2x	O1	1.961(2)
	2x	O2	2.023(2)		2x	O2	1.987(2)
				*M*2	2x	O1	2.033(2)
					2x	O2	2.053(2)
					2x	O3	2.070(2)

Table SI5. Results of BVS calculation for the atoms in the structure of Y_2NiTiO_6 -orthorhombic (*Pbmn*) and Y_2NiTiO_6-monoclinic (*P2₁/n*) perovskite.

Atom	Coordination	Average bond length, Å	Valence	Bond-valence sum
		Y_2NiTiO_6 -orthorhombic (*Pbmn*)		
O1	4.00	2.0839	-2.000	2.058
O2	5.00	2.2169	-2.000	1.890
Y	8.00	2.4227	3.000	2.966
0.482(8)Ti	6.00	2.0131	4.000	3.514
0.518(8)Ni	6.00	2.0131	2.000	2.274
		Y_2NiTiO_6-monoclinic (*$P2_1/n$*)		
O1	4.00	2.0838	-2.000	2.079
O2	5.00	2.2195	-2.000	1.908
O3	5.00	2.2099	-2.000	1.954
0.996(4)Y	8.00	2.4234	3.000	2.955
0.91(1)Ti	6.00	1.9682	4.000	3.968
0.09(1)Ni	6.00	1.9682	2.000	2.568
0.90(2)Ni	6.00	2.0519	4.000	2.049
0.10(2)Ti	6.00	2.0519	2.000	3.165

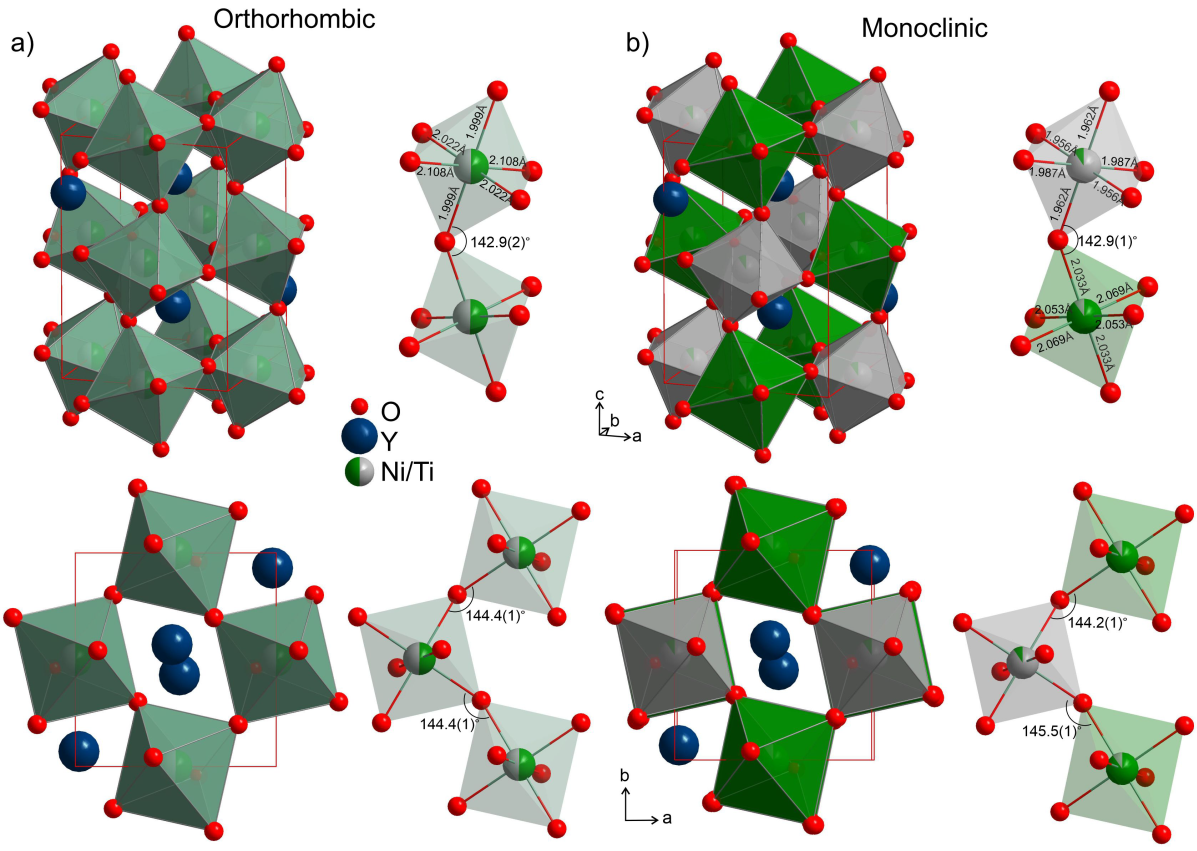

Figure SI1. Structures of Y_2NiTiO_6 perovskites projected along the *c*-axis (top) and *ab*-plane (bottom). a) Disordered Y_2NiTiO_6-orthorhombic perovskite (*Pbmn*). b) Mostly ordered Y_2NiTiO_6-monoclinic perovskite (*$P2_1/n$*). For the Ni/Ti@O_6 octahedra, relevant interatomic distances and angles are highlighted to showcase change of the coordination environments with ordering. Yttrium, nickel, titanium and oxygen atoms are drawn as dark-blue, green, grey, and red spheres, respectively.

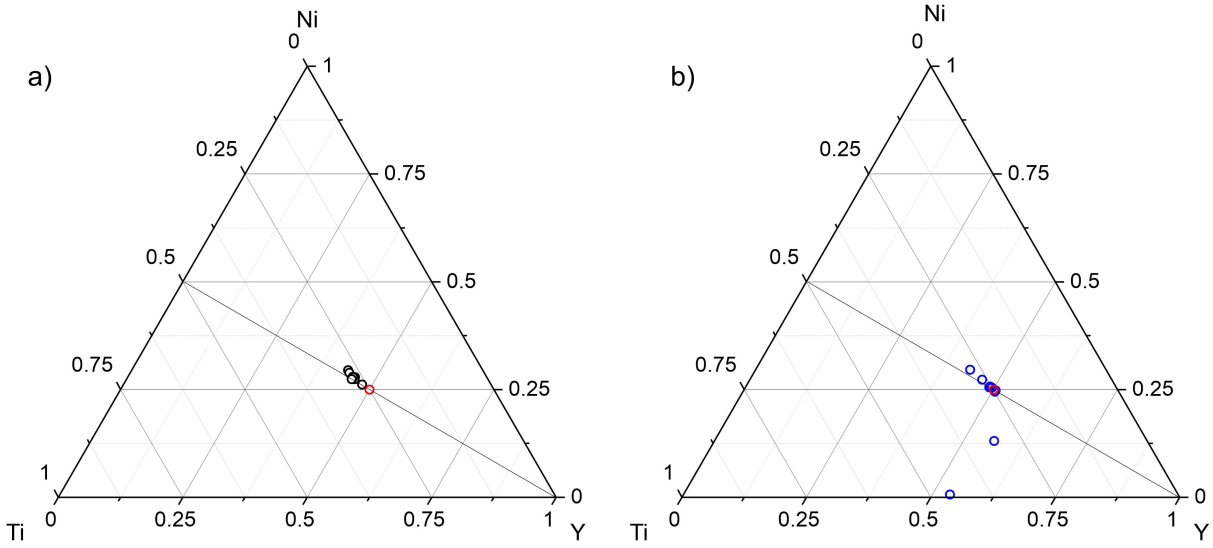

Figure SI2. EDX of the Y_2NiTiO_6 samples: a) annealed at 1570 K with mostly monoclinic phase; b) annealed at 1730 K with mostly orthorhombic phase.

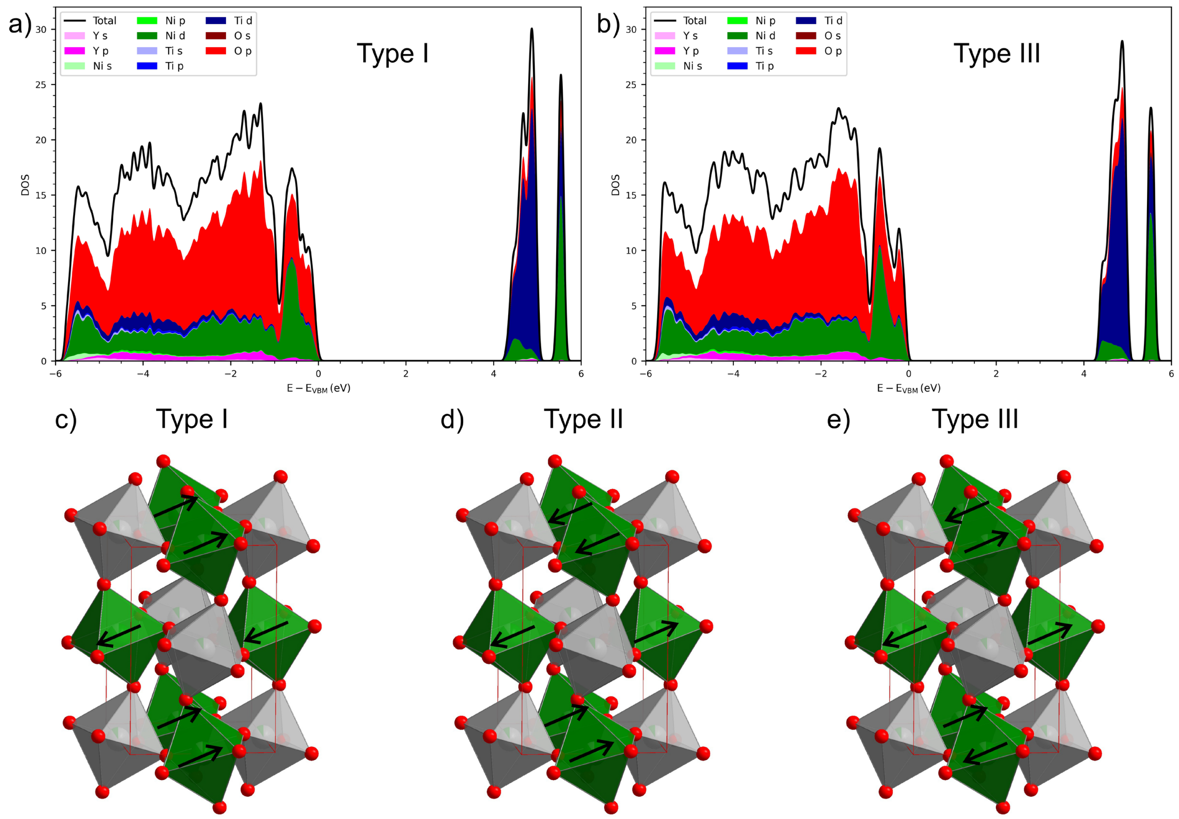

Figure SI3. Partial density of states of Type I (a) and Type III (b) anti-ferromagnetically ordered Y_2NiTiO_6 in the fully site-ordered $P2_1/n$ structure. Type I, II, and III magnetic ordering in double perovskites is shown in c-d.

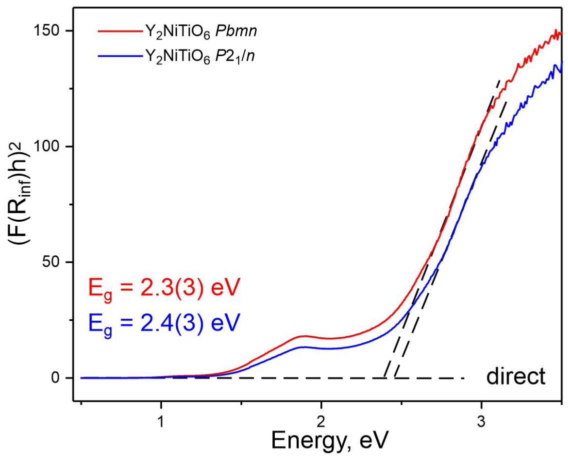

Figure SI4. UV-vis spectra of orthorhombic (red) and monoclinic (blue) Y_2NiTiO_6 perovskites used to determine the band gap using Tauc plot.

Table SI6. Fit parameters for relative absorption spectra (Figure 4, *b* and *c*) contribution to two Gaussian functions, an exponentially expressed Urbach tail, and a power-law dependant indirect band edge.

	Y_2NiTiO_6-orthorhombic (*Pbmn*)	Y_2NiTiO_6-monoclinic ($P2_1/n$)
X_g	2.16(1)	2.239(7)
n	0.5	0.5
A	3.78	3.7
A_1	0.009(4)	0.013(4)
X_u	–0.45(3)	–0.50(3)
y_0	–0.02(3)	–0.01(2)
X_c	2.4(3)	2.5(3)
A_2	1.75(4)	1.43(3)
A_3	0.56(2)	0.42(1)
x_a	1.779(3)	1.791(3)
x_b	1.063(2)	1.060(2)
w_1	0.317(4)	0.315(4)
w_2	0.167(4)	0.149(4)
Adj. R-Square	0.99897	0.99871

Fitting to:

if $(x > X_c)$

$$y = y_0 + A^{(x-X_g)^n} + A_2 e^{-0.5\times((x-x_a)/w_1)^2} + A_3 e^{-0.5\times((x-x_b)/w_2)^2}$$

if $(x < X_c)$

$$y = y_0 + A_1 e^{-x/X_u} + A_2 e^{-0.5\times((x-x_a)/w_1)^2} + A_3 e^{-0.5\times((x-x_b)/w_2)^2}$$

if $(x = X_c)$

$$A = \frac{\left(A_1 e^{\left(-\frac{x}{X_u}\right)}\right)}{(x - X_g)^n}$$

Where X_c is the matching point between the Urbach tail and the indirect absorption edge; X_g is the position of the absorption edge; X_u is the decay constant for the Urbach tail; x_a and x_b are the centres of the two Gaussians; w_1 and w_2 are the widths of the two Gaussians; A, A_1, A_2 and A_3 determine the heights of the various contributions; and y_0 is a constant background.[17]

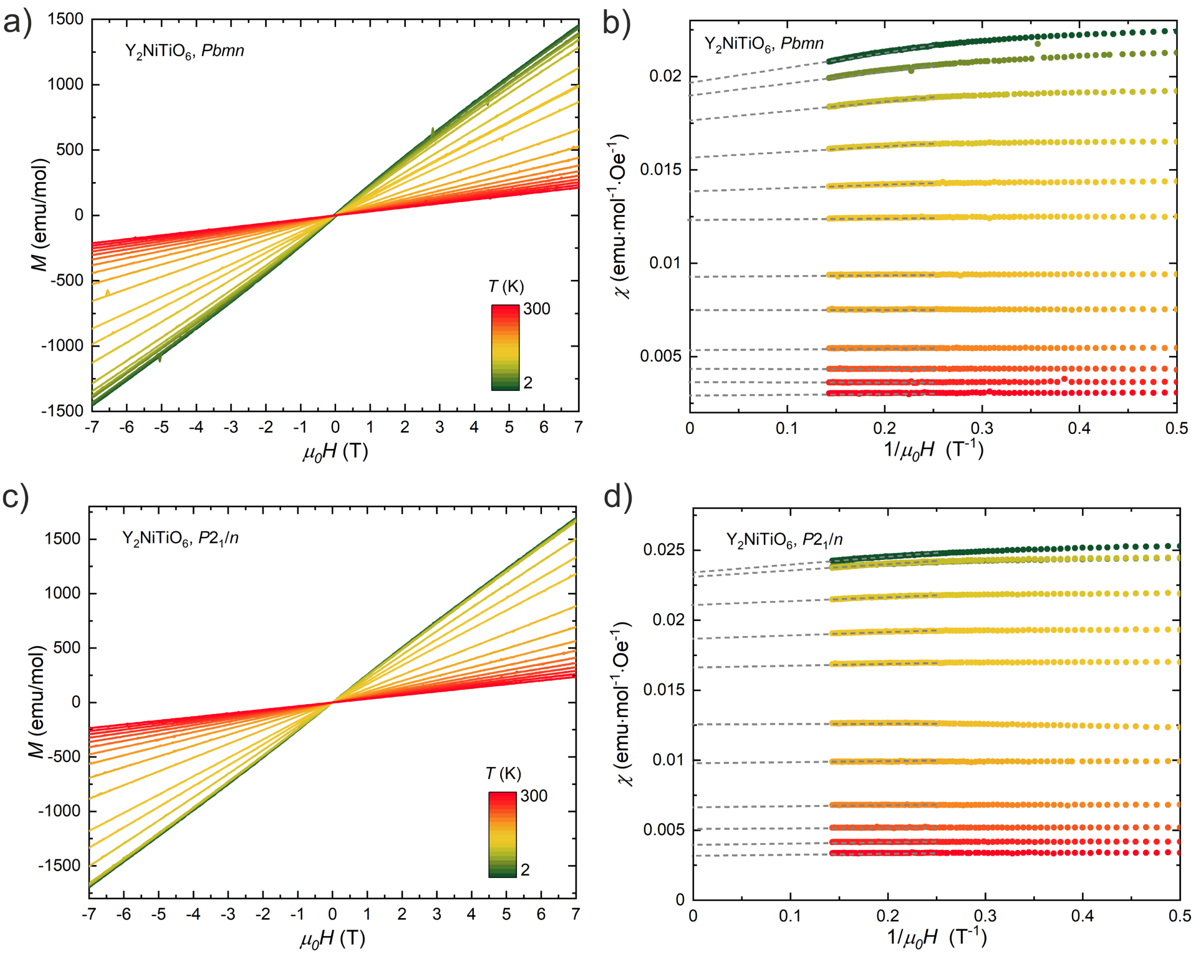

Figure SI5. Magnetic hysteresis loops at various temperatures for a) orthorhombic and c) monoclinic Y_2NiTiO_6, respectively. χ versus $1/\mu_0H$ plot to extract the correct magnetic susceptibility χ_{corr} via the Honda-Owen method for b) orthorhombic and d) monoclinic Y_2NiTiO_6, respectively. The dashed lines are the linear fits for the data between 4 – 7 T, and the resulting *y*-intercepts represent the χ_{corr}.

References:

(1) Cai, P.; Qin, L.; Chen, C.; Wang, J.; Bi, S.; Kim, S. I.; Huang, Y.; Seo, H. J. Optical Thermometry Based on Vibration Sidebands in Y_2MgTiO_6:Mn^{4+} Double Perovskite. *Inorg. Chem.* **2018**, *57* (6), 3073–3081.

(2) Godzhieva, O. V.; Porotnikov, N. V.; Petrov, K. I. A Physicochemical Study of Compounds Based on Zinc, Titanium, and Lanthanide Oxides. *Russ J Inorg Chem* **1987**, *32*, 1676–1677.

(3) Sederholm, L.; Tiittanen, T.; Karppinen, M. High-Pressure Stabilisation of *R* = Y Member of R_2CuTiO_6 Double Perovskite Series. *J. Solid State Chem.* **2023**, *317*, 123646.

(4) Kayser, P.; Alonso, J. A.; Muñoz, A.; Fernández-Díaz, M. T. Structural and Magnetic Characterization of the Double Perovskites R_2NiRuO_6 (*R* = Pr-Er): A Neutron Diffraction Study. *Acta Mater.* **2017**, *126*, 114–123.

(5) Mouallem-Bahout, M.; Roisnel, T.; André, G.; Gutierrez, D.; Moure, C.; Peña, O. Nuclear and Magnetic Order in Y(Ni,Mn)O_3 Manganites by Neutron Powder Diffraction. *Solid State Commun.* **2004**, *129* (4), 255–260.

(6) Deng, Z.; Kang, C.-J.; Croft, M.; Li, W.; Shen, X.; Zhao, J.; Yu, R.; Jin, C.; Kotliar, G.; Liu, S.; Tyson, T. A.; Tappero, R.; Greenblatt, M. A Pressure-Induced Inverse Order–Disorder Transition in Double Perovskites. *Angew. Chem. Int. Ed.* **2020**, *59* (21), 8240–8246.

(7) Blasco, J.; García, J.; Subías, G.; Stankiewicz, J.; Rodríguez-Velamazán, J. A.; Ritter, C.; García-Muñoz, J. L.; Fauth, F. Magnetoelectric and Structural Properties of Y_2CoMnO_6: The Role of Antisite Defects. *Phys. Rev. B* **2016**, *93* (21), 214401.

(8) Das, I.; Chanda, S.; Saha, S.; Dutta, A.; Banerjee, S.; Bandyopadhyay, S.; Sinha, T. P. Electronic Structure and Transport Properties of Antiferromagnetic Double Perovskite Y_2AlCrO_6. *RSC Adv.* **2016**, *6* (84), 80415–80423.

(9) Yang, L.; Duanmu, Q.; Hao, L.; Zhang, Z.; Wang, X.; Wei, Y.; Zhu, H. Ferrimagnetism and Possible Double Perovskite Structure in Half Cr-Doped $YMn_{0.5}Cr_{0.5}O_3$. *J. Alloys Compd.* **2013**, *570*, 41–45.

(10) Villars, P.; Cenzual, K. Pearson's Crystal Data: Crystal Structure Database for Inorganic Compounds (Release 2023/24), 2023, ASM International®, Materials Park, Ohio (USA).

(11) Nuvula, S.; Sagar, T. V.; Valluri, D. K.; Sai Prasad, P. S. Selective Substitution of Ni by Ti in $LaNiO_3$ Perovskites: A Parameter Governing the Oxy-Carbon Dioxide Reforming of Methane. *Int. J. Hydrog. Energy* **2018**, *43* (8), 4136–4142.

(12) Souza, M. M. V. M.; Maza, A.; Tuza, P. V. X-Ray Powder Diffraction Data of $LaNi_{0.5}Ti_{0.45}Co_{0.05}O_3$, $LaNi_{0.45}Co_{0.05}Ti_{0.5}O_3$, and $LaNi_{0.5}Ti_{0.5}O_3$ Perovskites. *Powder Diffr.* **2021**, *36* (1), 29–34.

(13) Rodríguez, E.; López, M. L.; Campo, J.; Veiga, M. L.; Pico, C. Crystal and Magnetic Structure of the Perovskites La_2MTiO_6(*M* = Co, Ni). *J. Mater. Chem.* **2002**, *12* (9), 2798–2802.

(14) Pérez-Flores, J. C.; Pérez-Coll, D.; García-Martín, S.; Ritter, C.; Mather, G. C.; Canales-Vázquez, J.; Gálvez-Sánchez, M.; García-Alvarado, F.; Amador, U. *A*- and *B*-Site Ordering in the *A*-Cation-Deficient Perovskite Series $La_{2-x}NiTiO_{6-\delta}$ ($0 \le x < 0.20$) and Evaluation as Potential Cathodes for Solid Oxide Fuel Cells. *Chem. Mater.* **2013**, *25* (12), 2484–2494.

(15) Rodríguez, E.; Álvarez, I.; López, M. L.; Veiga, M. L.; Pico, C. Structural, Electronic, and Magnetic Characterization of the Perovskite $LaNi_{1-x}Ti_xO_3$ ($0 \le x \le 12$). *J. Solid State Chem.* **1999**, *148* (2), 479–486.

(16) Karaphun, A.; Hunpratub, S.; Phokha, S.; Putjuso, T.; Swatsitang, Ekaphan. Characterization and Magnetic Properties of $SrTi_{1-x}Ni_xO_3$ Nanoparticles Prepared by Hydrothermal Method. *Phys. B Condens. Matter* **2017**, *504*, 31–38.

(17) Makuła, P.; Pacia, M.; Macyk, W. How To Correctly Determine the Band Gap Energy of Modified Semiconductor Photocatalysts Based on UV–Vis Spectra. *J Phys Chem Lett* **2018**, *9* (23), 6814–6817.
